# Novel CDKL5 mutations were found in patients in China: retrospective investigation in cases of CDKL5-related disorders

**DOI:** 10.1186/s13052-020-0775-y

**Published:** 2020-02-28

**Authors:** Yumei Yan, Dake He, Jing Wu, Ruolin Hou, Kun Sun, Ling Li

**Affiliations:** 1 Department of Pediatric Neurology, Xinhua Hospital Affiliated to Shanghai Jiaotong University, School of Medicine, Shanghai, 200092 China; 2 Xinhua Hospital Affiliated to Shanghai Jiaotong University, School of Medicine, Shanghai, 200092 China

**Keywords:** de novo, Mutations, CDKL5, CDKL5-related disorders (CDD), Intellectual disability, Epileptic encephalopathy

## Abstract

**Objective:**

CDKL5-related disorders (CDD) is an epileptic encephalopathy resulted of gene mutations of CDKL5. This study aimed to explore the development process of CDD and to expand its mutation spectrum.

**Methods:**

Clinic datawas collected about three infantile epileptic encephalopathy cases diagnosed at Xinhua Hospital Affiliated to Shanghai Jiaotong University, School of Medicine. Next generation sequencing technology was used to find three de novo mutations of CDKL5. We searched published literatures about CDKL5 in pubmed and made an analysis about our clinic data and the related literatures.

**Results:**

The three patients were all girls. Their average onset age of seizures was around 2 months, and all of them have intractable epileptic seizures, severe intellectual disability, and hypotension. Among them, two presented infantile spasm and high arrhythmia in EEG, and the other manifested clonic seizure and broad epileptiform discharge in EEG. Extracerebral space widening in cranial MRIs was demonstrated in two cases. Visual evoked potential was abnormal in two cases. Seizures were resistant to all kinds of antiepileptic drugs (AEDs). Gene tests showed three de novo mutations of CDKL5: one was a truncated mutation (c.2254A > T,P.R752X, stop279), which was pathogenic according to the ACMG guide, the other two were missense mutations (c.377G > T,p.Cys126Phe) and a frameshift mutation (c.362-362insG(p.Ala122GlyfsTer7), which were likely pathogenic according to the ACMG.

**Conclusions:**

All three de novo mutations are first reported. Based on the combined related literature and the manifestations observed, we diagnosed the three children as CDKL5-related disorders, and concluded that the de novo CDKL5 mutations are the reason for their epilepsy.

## Introduction

Cyclin-dependent kinase-like 5 (CDKL5, OMIM 300203, also known as serine/threonine kinase 9, STK9) belongs to the serine/threonine kinase protein family. It is composed of a conserved N-terminal catalytic domain and a long variable C-terminal extension, which may have a regulatory role [1]. It is broadly expressed in the brain, and its mutations can result in severe nervous system disorders called CDKL5-related disorders (CDD) [2–4]. These disorders include early-onset intractable epileptic encephalopathy, severe intellectual disability, hypotension, and vision impairment, with an incidence of about one in 40,000 to 60,000 live births [5, 6]. So far more than 800 mutations of CDKL5 have been collected in Clinvar, and half of them have been classified as pathogenic or likely pathogenic. Here we reported three children diagnosed with CDD in our hospital and analyzed their data.

## Methods

Patients were chosen from children who were diagnosed with epileptic encephalopathy and received therapy at our hospital. All of them signed the informed consent. Clinic data containing their medical history, blood tests, cranial MRI, electrophysiological changes, therapy procedures and prognosis were collected. Next generation sequencing technology was used to determine the CDKL5 mutations after obtaining their parents’ permission. By collecting studies related to CDKL5 in PubMed, we performed a retrospective analysis of their data.

## Results

The patients were all girls. Except Patient 1 whose mother had a history of unhealthy pregnancies with three drug-induced abortions before her and diabetes mellitus during early pregnancy, the other two were the first pregnancy of their mothers. All the patients were delivered full-term, without any history of suffocation rescue. However, patient 2 showed a smaller gestational age with a birth weight of 2450 g. There was no record of defects in their family history. The average age of onset in the three girls was 2 months.

**Fig. 1:**
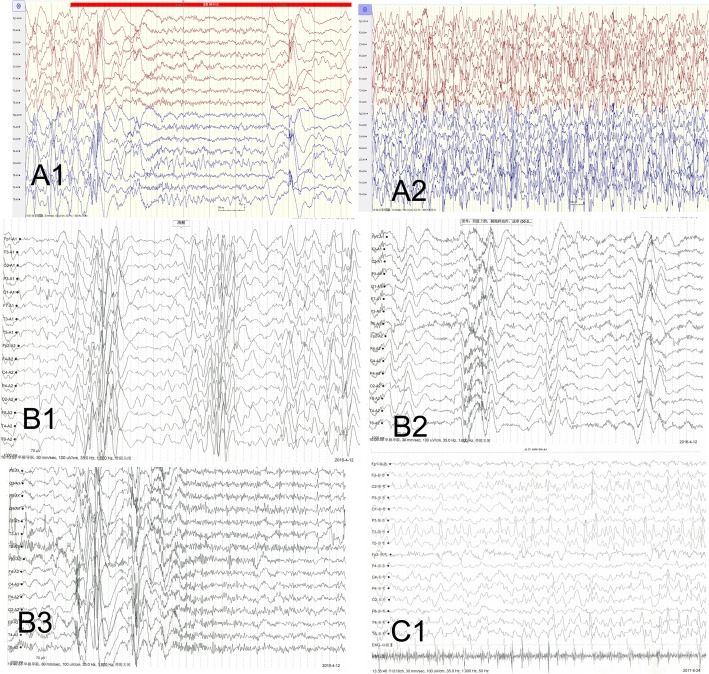
EEGs of the three girls. **A1** shows the spasm of patient 1 at 18 m. **A2** is the episodic electroencephalogram of patient 1 as spikes, Spikes and slow waves were observed in all leads and the girls presented spastic seizures; **B1** shows the hypsarrhythmia of patient 2 at 8 m. **B2**/**B3** shows seizures of spasms followed by tonic-clonic seizures with EEG; **C1** shows the spike and spike-slow waves in multiple leads of patient 3

Focal secondary generalized tonic-clonic seizures were first presented in patient 1 at 2 months, and were verified by video-EEG. Later, the seizures transformed to infantile spasm at 18 months, as shows in Fig. 1-A1, A2. OCX, VPA, TPM, ACTH, MP were given successively, but no effects were observed. Moreover, she presented hypotension in her four limbs and her intellectual disability became obvious gradually. She raised her head at 3 months, and turned over at 9 months. Although she is 2 years old now, she can only sit alone for a while. Her eyes were unable to chase any objects. She cannot speak any word and react for any communication at present.

Patient 2 first displayed general clonic seizures at 2 months and developed infantile spasms 1 month later. EEG presented hypsarrhythmia in the interictal phase and a series of spike-slow wave discharges when the seizures occurred leading to a diagnosis of infantile spasms, as shows in Fig. 1-B1, B2, B3. VPA, NZP, TPM, ACTH, and MP were successively given to control the seizures. Except ACTH, no other AEDs were useful. However, several months after ACTH treatment, her seizures relapsed. Her intellectual disability is severe. She is now 4 years old; she can only sit for a while, and never stands or walks. She can only pronounce Ah, has poor eye contact and cannot recognize her parents. She can cry when she wants to visit the toilet. Hypotension and hand failure were also observed in this patient.

Patient 3 presented with generalized clonic seizures several times a day when she was 80 days old and synchronized electroencephalogram displayed broad epileptiform discharges, as shows in Fig. 1-C1. Levetiracetam and ACTH did not show any effects. Intellectual disability was obvious as she does not make any eye contact. She is now 2 years old, but she cannot raise her head, or sit alone; she cannot pronounce any sound and move her hand actively. The detailed clinical characteristics of all three patients are listed in Table 1.

**Table 1:** Clinical data of the three patients

Patient	Patient 1	Patient 2	Patient 3
Current age	2 years	4 years	2 years
Age of onset	2 months	2 months	80 days
Gender	F	F	F
Personal history	G5P2, full term and smooth delivery, BW 3500 g	G1P1, full term and smooth delivery, BW 2450 g,	G1P1, full term and smooth delivery, BW 3395 g
Pregnancy history	Diabetes mellitus with good control of blood sugar	Normal	Upper respiratory tract infection during early pregnancy but without any medication
Familial history	Normal	Normal	Normal
Epilepsy	PS, TCS, SS	TCS, TS, CS, SS	TCS, CS
EEG	F, H	H, M	M
MRI	Normal	Wide anterior sulcus of bilateral frontotemporal lobe	Wide anterior sulcus of bilateral frontotemporal lobe
AED	VPA, TPM, OCX, ACTH, MP	VPA, NZ, TPM, ACTH, MP	LEV, ACTH
VEP	abnormal	abnormal	normal
Intellectual disability	Severe	Severe	Severe
Seizure control	Undone	Undone	Undone

*PS* focal seizure, *TS* tonic seizure, *TCS* tonic-clonic seizure, *CS* clonic seizure, *SS* spastic seizure, *MS* myoclonic seizure, *N* normal, *H* high arrhythmia, *F* focal discharge, *M* multifocal discharge, *PHE* phenobarbital, *LEV* levetiracetam, *VPA* sodium valproate, *TPM* topiramate, *VBG* aminohexenoic acid, *KD* ketogenic diet, *NZP* nitrodiazepam, *LTG* lamotrigine, *OXC* oxcarbazepine, *MP* Methylprednisolone, *DXM* Dexamethasone, *ACTH* adrenocorticotropin, *CBZ* carbamazepine

**Fig. 2:**
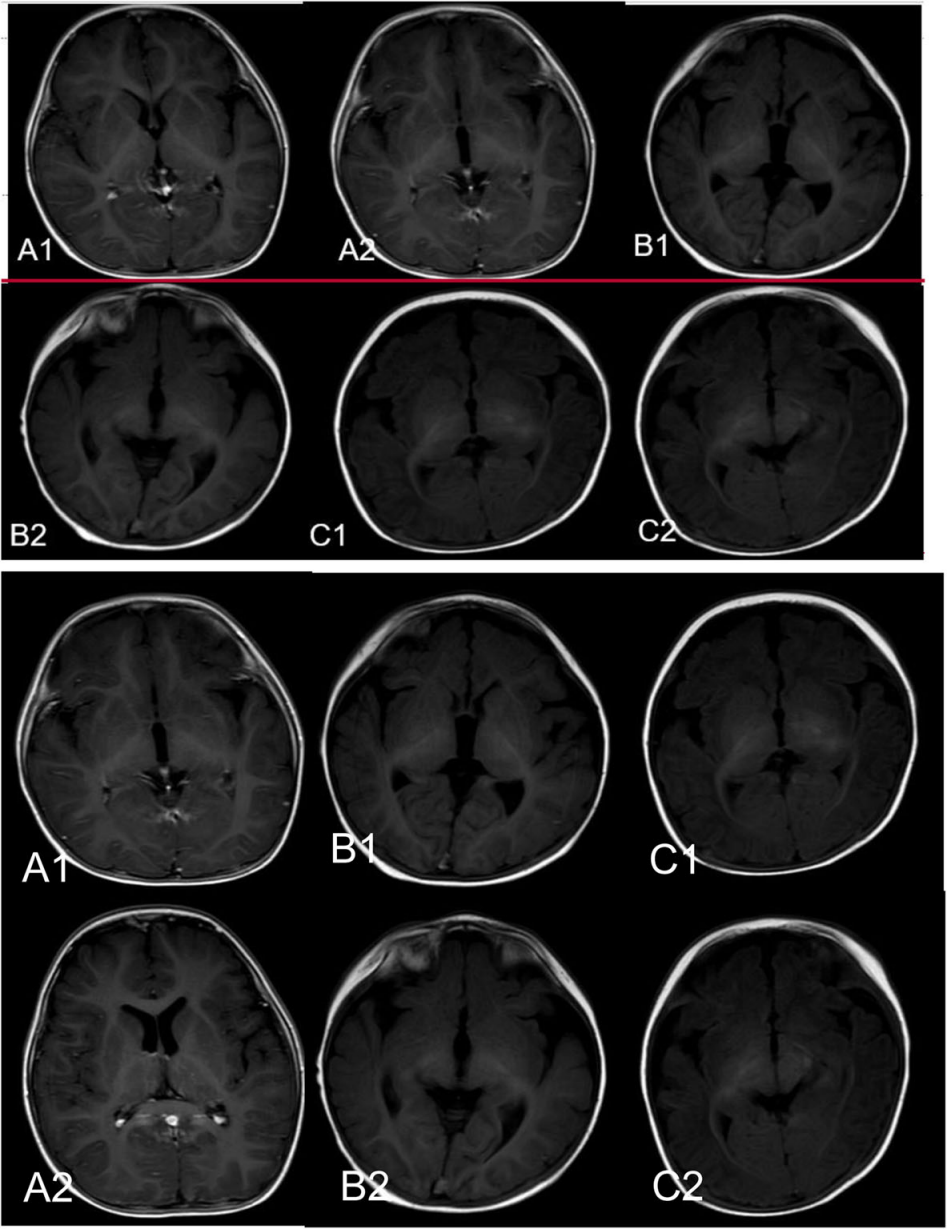
Shows the cranial MRIs of the three girls. **A1** and **A2** are the normal images of patient1. **B1**/**B2** and **C1**/**C2** show the wider anterior sulcus of the bilateral frontotemporal lobe in patient 2 and patient 3

**Fig. 3:**
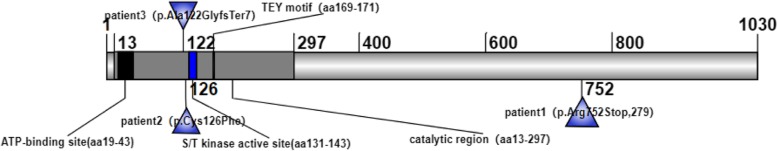
shows a schematic representation of the CDKL5 protein and its main functional region. The mutations reported in our paper are also marked here

During hospitalization, a series of tests were completed, including blood routine, biochemical, etiological examination, inflammatory factors, cerebrospinal fluid examination, cranial MRI, electroencephalogram (EEG), auditory/visual evoked potential (AEP/VEP), and so on. Cerebrospinal fluid (CSF) and blood tests were normal in all three patients. Except for CMV infection in patient 1, We did not find any infectious factors. Wider anterior sulcus of bilateral frontotemporal lobes were found in patient 2 and 3, but this was not specific. The cranial MRIs are enclosed in Fig. 2. Moreover, the results of Visual evoked potential (VEP) implied prolonged bilateral P100 values, poor repetition and differentiation in the bilateral eyes among patient 1 and 2, which was in agreement with their lack of eye contact. With their parents’ permission, we performed gene tests. Three de novo mutations of CDKL5 were found, and their locations are marked in the CDKL5 gene ideograph in Fig. 3. The gene details are displayed in Table 2. Based on the American Society of Medical Genetics (ACMG), the mutations were subgrouped as pathogenic or probably pathogenic.

**Table 2:** The CDKL5 mutations in the three girls

	Patient 1	Patient 2	Patient 3
Gene mutation	c.2254A > T	c.377G > T	c.362_363insG
protein	p.Arg752Stop,279	p.Cys126Phe	p.Ala122GlyfsTer7
father	wild type	wild type	wild type
mother	wild type	wild type	wild type
Mutation style	Heterozygous, truncation mutation	Heterozygous, missense mutation	Heterozygous, frameshift mutation
ACMG degree	Pathogenic	Likely pathogenic	Likely pathogenic

## Discussion

CDKL5 protein is widely expressed in the brain and plays important roles in cell proliferation, neuronal migration, axonal outgrowth, dendritic morphogenesis, and synapse development [7, 8]. Typical clinical characters of CDKL5-related disorders (CDD) include infantile-onset refractory epilepsy, hypotonia, developmental delay, intellectual disability, and visual impairment [2–4, 9]. Seizures are always onset in the first 3 months of life, and infantile spasms occur in about 81% percent of patients [10]. Other seizure types include focal and generalized seizures with spasms, tonic and tonic-clonic seizures, or a mixture of both, and autonomic changes such as irregular respiration, apneas, or hyperventilation are also intermixed [9, 10]. EEG may be normal at first in some patients, but subsequent EEGs show high arrhythmia, focal spike discharge, multifocal or all lead spikes, spike-slow wave discharge [10, 11]. Seizures are always resistant to various kinds of AEDs and corticosteroids. Ketogenic diet (KD) were also tried, but side effects and poor long-term efficacy remain significant barriers [12–14]; Vagus nerve stimulation (VNS) was also applied to control the intractable epilepsy associated with CDD, and though there are few reports and cases, the results were found satisfactory [14–16]. Generally CDD are divided into 3 stages according to the disease process [17]: (1) early onset, which is pharmaco-responsive at times, (2) epileptic encephalopathy, and (3) refractory multifocal and myoclonic epilepsy. The honeymoon period always occurs in childhood. Among our patients, two presented infantile spasms: patient 1 first displayed generalized tonic clonic seizures secondary to focal seizures, and progressed to IS at 18 months. Patient 2 first presented clonic seizures at 2 months and quickly progressed to IS 1 month later. Although various kinds of AEDs and ACTH were administered, the seizures were unsatisfactorily controlled. The parents of patient 2 and patient 3 stopped all the AEDs simply because of unsatisfactory control, but the seizures still stopped at later times, which maybe just reached their honeymoon period. Patient 3 displayed clonic seizures with involvement of upper limbs, lower limbs, or both. The frequency reached 6 times a day. Levetiracetam and Corticotropin were given to control the seizure but finally failed.

Intellectual disability occurs in almost every child with CDD [2–4, 9–11], and was also observed in our patients. At present, two patients are 2 years old, but can only sit alone for a while, and can never walk or say a word; there is no communications with others, regardless of eye contact or sound stimuli, and they show no movement of their hands. The other patient is 4 years old, she can cry or pronounce Ah when she wants to visit the toilet, but cannot go by herself. She can sit alone for a while but can never stand or walk. Other characters of CDD such as hypotonia and vision impairment were also observed in our patients. All three patients showed no eye contacts and destruction in the visual pathway was demonstrated by VEP in two patients. MRI was non-specific, it can be normal or can show non-specific abnormalities, just as in our patients.

The CDKL5 gene is located in the short arm of the X-chromosome at position 22 (Xp22), and contains 20 coding exons; its importance was emphasized first when Montini et al. first discovered it in patients in 1998 [1]. Mutations of CDKL5 include missense mutation, truncation mutation, frameshift mutation, and so on [18]. Its first mutations were found in 2003 by Kalscheuer et al. [19]. So far more than 100 pathogenic mutations have been reported. The clinical severity is associated with the location and type of mutations; for example, missense mutations located in the catalytic domain of CDKL5 could exhibit more severe phenotypes compared to patients carrying other CDKL5 mutations [8], but the exact genotype-phenotype correlation still remains obscure. In our patients, the three mutations are all de novo, as no mutations were found in their parents, and there were no reports previously. Although the 126 AA location change to Trp(c.378C > G (p.Cys126Trp)) of CDKL5 can be observed in other infantile spasm patients in Clinvar, in our patient 2, the 377 base mutation (c.377G > T) translated to 126 Phe (p.Cys126Phe), is the first report to our knowledge. The mutation occurred in the kinase domain, and AGMG categorized it as likely pathogenic. The mutation in Patient 1 is a truncation mutation in exon 15(c.2254A > T, p.Arg752Stop,279) in the regulatory C-terminal, meaning the protein after 752 aa cannot be translated. The variation was classified as pathogenic according to the AGMG. Patient 3 showed a frameshift mutation c.362-363insG(p.Ala122GlyfsTer7), with a G base inserted at position 363 resulting in AA changes after 122Ala; moreover, it was terminated 7 AA later. The mutation occurred in the conserved N-terminal catalytic domain, and is classified as likely pathogenic according to the AGMG.

The CDKL5 inheritance pattern is XD, and almost all patients reported are girls. The reason may be that boys carrying the mutation are miscarried or die before diagnosis. Recently there have been several papers reporting the CDKL5 mutation in both genders [20–22]. Liang JS, et al. at first [21], did not find any difference among the genders but recently concluded that male children demonstrate more severe phenotypes, a higher frequency of infantile spasms and brain atrophy, whereas female children often exhibit an atypical Rett syndrome with EoEE [20], just as concluded by Elia M [22]. As all our patients were girls, we cannot make any comparisons between genders. In future, we will collect male patients with CDKL5 mutations and try to determine the gender differences.

## Conclusion

CDKL5 protein is an important protein expressed in the brain and its mutation results CDKL5-related disorders (CDD) representing typical characters including infantile-onset refractory epilepsy, hypotonia, developmental delay, intellectual disability, and visual impairment. Its inheritance type is XD and most patients are girls. In our three patients, the clinical manifestations coincided with CDDs and their de novo mutations were the reasons for their epilepsy. Next we will collect more data to determine the gender differences.

## Data Availability

All data generated or analysed during this study are available from the corresponding author on reasonable request.

## Abbreviations

ACMG: American Society of Medical Genetics
AED: Antiepileptic drugs
AEP/VEP: Auditory/visual evoked potential
CDD: CDKL5-related disorders
CSF: Cerebrospinal fluid
EEG: Electroencephalogram
KG: Ketogenic diet
VNS: Vagus nerve stimulation
